# Municipal Outsourcing and Animal Welfare Outcomes in Contracted Dog Shelters: A 10-Year Retrospective Policy Evaluation in Korea

**DOI:** 10.3390/ani16172657

**Published:** 2026-08-24

**Authors:** Byeong-Cheol Song

**Affiliations:** Department of Agriculture and Livestock, Yeongyang County Office, 37 Guncheong-gil, Yeongyang-eup, Yeongyang-gun 36537, Republic of Korea; sbc07016@korea.kr

**Keywords:** animal shelter, dog, public outsourcing, operator type, live release, euthanasia, municipal governance, public policy, veterinary public health

## Abstract

Municipal dog shelters are often grouped as outsourced facilities despite operator differences. We analyzed 33,875 dog admissions and 90 municipality-year observations from 2016 to 2025 across nine Korean municipalities, with three per operator category; operator type was fixed and nested within municipality. Among 32,838 known outcomes, live-release rates were 69.1%, 58.1%, and 40.0%, and euthanasia rates were 14.0%, 22.8%, and 42.5% in corporation-operated, veterinary hospital-operated, and individually operated shelters, respectively. The corporation live-release estimate ranged from 62.6% to 72.0% under alternative classifications of unresolved records. Small-sample cluster-robust comparisons were imprecise: the individually versus corporation-operated euthanasia odds ratio was 4.72 (95% confidence interval, 0.40–56.35), and every operator interval included 1.0. Contextual adjustment yielded odds ratios of 0.97 for both outcomes for veterinary hospital- versus corporation-operated shelters. Among 33,836 admissions with common 180-day follow-up, live-release rates were 59.9%, 53.0%, and 39.8%, and euthanasia rates were 9.1%, 16.7%, and 42.0%; annual odds ratios were 1.05 (0.93–1.19) and 0.90 (0.78–1.05), respectively. Results describe operator-linked municipal systems, not causal effects. Standardized follow-up, complete reporting, capacity monitoring, and larger prospective studies are needed.

## Simple Summary

Local governments often hire outside providers to run municipal dog shelters, but different types of providers are commonly treated as one group. We examined dog admission records from 2016 to 2025 in nine municipalities in Gyeongsangbuk-do, Korea: three used corporations, three used veterinary hospitals, and three used individual operators. Among dogs with a recorded final outcome, corporation-run shelters had the largest share of dogs leaving alive via return to their owner, adoption, or transfer. Veterinary hospital-run shelters had the largest share of dogs returned to owners, whereas individually run shelters had the largest share euthanized. We also compared dogs that could all be followed for the same 180 days after admission, preventing recently admitted dogs from having less time to reach an outcome. By day 180, the shares leaving alive were 59.9%, 53.0%, and 39.8%, and the shares euthanized were 9.1%, 16.7%, and 42.0%, respectively. These differences do not prove that provider type caused better or worse outcomes because the municipalities also differed in resources, policies, and operating conditions. More complete records, routine monitoring of shelter capacity, and studies involving more municipalities are needed to support fair contracting decisions and improve dog welfare.

## 1. Introduction

Municipally designated animal protection centers constitute an important interface between veterinary public health, local administration, and companion-animal welfare. In Korea, current animal-protection legislation and official administrative reports provide the framework for rescue, protection, return to owner, rehoming, natural mortality, and euthanasia of lost and abandoned animals [1,2]. Operational outcomes are therefore both administrative indicators and welfare-relevant measures that describe whether a dog leaves the shelter alive, how long it remains in care, and whether it experiences death or euthanasia while in the shelter [3,4].

Under the current Korean Animal Protection Act and its Enforcement Rule, local governments are responsible for rescuing and protecting lost or abandoned animals, checking registration status and notifying owners when applicable, publicly announcing protective measures, returning or rehoming animals, and ensuring that humane disposition follows prescribed requirements [2]. The statutory pathway includes a public-notice period of at least seven days and permits municipal acquisition of ownership when the owner remains unknown 10 days after public announcement [2].

Previous shelter studies have shown that dog characteristics, including age, body size, breed designation, health status, behavior, and other phenotypic or intake-related factors, can influence adoption, return to owner, euthanasia, length of stay, and shelter resource allocation [5,6,7,8,9]. Other studies have emphasized that sheltering conditions can affect canine stress, behavior, immune function, and welfare, particularly when animals experience crowding, noise, social isolation, restricted space, limited enrichment, prolonged confinement, or long-term sheltering [10,11,12,13,14,15,16,17,18]. Standardized behavioral assessments may also be relevant to adoption suitability and post-adoption outcomes, although such tools require careful validation before being used as deterministic disposition criteria [19,20]. These findings suggest that shelter outcomes are produced by both dog-level case mix and the operational environment in which care is delivered.

Contracted animal protection centers may be operated by non-profit corporations or animal welfare organizations, veterinary medical facilities, or natural persons. Nevertheless, official and research summaries often treat contracted shelters as one homogeneous administrative category. Few longitudinal studies have examined whether this broad label conceals descriptive differences among operator-linked municipal systems while also accounting for repeated municipality observations, unequal intake composition, and unresolved outcomes. Public-management and governance scholarship indicates that outsourced service outcomes may reflect contract design, institutional alignment, stewardship relationships, stakeholder networks, and co-production rather than provider legal form alone [21,22,23,24,25,26].

This study therefore compared live release, adoption, return to owner, natural mortality, euthanasia, and length of stay across three operator categories using 10 years of administrative records from nine municipalities in Gyeongsangbuk-do, Korea. The Operator category was assigned at the municipality level and remained unchanged throughout the study period. The estimand was consequently an ecological between-municipality association, not an individual dog effect or the effect of changing an operator within the same municipality.

The specific research gap was not whether one operator category is intrinsically superior but whether the broad outsourcing label conceals welfare-relevant patterns among different municipal service configurations. The study was designed to generate hypotheses for monitoring and prospective evaluation; it did not test causal mechanisms or the effectiveness of any contract standard or policy intervention.

## 2. Materials and Methods

### 2.1. Study Design and Setting

This study was designed as a retrospective ecological observational study using routinely collected shelter records and a municipality-year panel dataset. Reporting was structured primarily with reference to the Strengthening the Reporting of Observational Studies in Epidemiology (STROBE) statement and the STROBE-Vet statement [27,28]. Because the source records were administrative data, relevant items from the RECORD extension were additionally reviewed [29]. The study area comprised nine municipalities in Gyeongsangbuk-do, Republic of Korea, that operated municipally designated animal protection centers through private outsourcing contracts between 2016 and 2025.

Based on municipal operational records, contracted shelter operators were classified into three mutually exclusive categories: corporation-operated, veterinary hospital-operated, and individually operated shelters. Corporation-operated shelters included centers operated by non-profit corporations or animal welfare organizations; veterinary hospital-operated shelters were operated by veterinary medical facilities; and individually operated shelters were managed by natural persons. The municipalities were Andong-si, Seongju-gun, and Chilgok-gun for the corporation-operated category; Pohang-si, Sangju-si, and Gyeongsan-si for the veterinary hospital-operated category; and Mungyeong-si, Uiseong-gun, and Goryeong-gun for the individually operated category. Each municipality remained in one operator category for all ten years. Operator category and municipality identity were therefore perfectly nested in this dataset: the 90 municipality-year rows represented repeated annual observations within only nine independent municipalities, with three municipalities per operator category.

### 2.2. Data Sources and Study Population

The primary data sources were individual-level administrative records of lost and abandoned animals and internal municipality-year operational records obtained from the nine selected municipalities. Individual-level records contained information on intake, animal characteristics, sheltering history, and final disposition. Municipality-year records contained information on operator type, maximum shelter capacity, staffing size, total annual budget, rescue-related budget, and veterinary care budget. The nine municipal datasets were initially extracted on 22 April 2026 and were last updated under a common data-closure date of 25 June 2026.

The study population was restricted to dogs admitted to the selected animal protection centers between 2016 and 2025. Cats and other animal species were excluded. The intake year was determined primarily from the rescue or admission date. When the rescue or admission date was unavailable, inconsistent, or ambiguous, the official report date was used as a secondary criterion. Records outside the target observation window and records with implausible or unresolvable dates were excluded from the primary analysis if they could not be reliably assigned to an intake year.

### 2.3. Exposure Variable and Operator Classification

The primary exposure was the contracted shelter operator category, defined as corporation-operated, veterinary hospital-operated, or individually operated. Corporation-operated shelters were used as the reference category to provide a common comparator; this choice did not imply that the reference category was a causal baseline or a uniformly superior model.

Operator classification was assigned at the municipality level from official operational records. Because operator category did not vary within municipalities, it was a time-invariant ecological exposure and was completely confounded with municipality identity at the between-municipality level in this sample. The data could not separate operator category from stable municipal characteristics such as geography, infrastructure, contract design, or long-standing resource conditions. This constraint informed the modeling strategy and the interpretation of all operator coefficients.

### 2.4. Outcome Definitions

The primary outcome was live release. Live release was defined as any confirmed final disposition in which a dog left the shelter alive, including adoption, return to owner (RTO), transfer to another facility or organization, release in a live state, or another documented live outcome. The live-release rate was calculated as the proportion of dogs with confirmed final dispositions that resulted in live release.

Secondary outcomes included adoption, RTO, transfer, natural mortality, euthanasia, length of stay (LOS), and unresolved or long-term sheltering status. Adoption was defined as placement with a new owner or household. RTO was defined as the return of a lost dog to its identified owner or caretaker. Transfer was defined as movement to another institution, organization, or facility while the dog was alive. Natural mortality comprised in-shelter deaths not explicitly coded as euthanasia, including records coded as natural death, death due to illness, unassisted death, or equivalent terminology. Euthanasia was defined strictly as cases explicitly recorded as euthanasia, humane destruction, or equivalent terminology indicating intentional humane killing. Cases not explicitly coded as euthanasia were not included in the euthanasia category.

For final-disposition outcome rates, the denominator was restricted to dogs with confirmed final outcomes. These analyses are therefore final-disposition analyses at the data-closure date, not complete lifetime outcome analyses. Dogs still in care were right-censored at data closure, while missing, ambiguous, or unclassifiable records were treated as unresolved; all were excluded from final-disposition denominators but retained for data-completeness analyses. LOS was calculated from admission to recorded final disposition and was restricted to records with valid dates; missing, negative, or logically inconsistent intervals were excluded from LOS-specific analyses. For the reviewer-requested fixed 180-day analysis, eligibility required intake on or before 27 December 2025, providing 180 days of potential follow-up by 25 June 2026. Live release, euthanasia, and natural mortality were classified when the documented event occurred on days 0–180 after intake. Events occurring after day 180 and records without a documented event by day 180 were coded as no event for the corresponding binary endpoint; admissions after the eligibility cutoff were excluded.

### 2.5. Covariates and Municipality-Year Operational Variables

Individual-level descriptors extracted from shelter records included age, sex, breed, body-size category or body weight, and health condition at intake, where available. Breed information was derived from free-text or categorical entries in the original administrative records and standardized for analytic use. Mixed-breed status was classified separately where the record permitted distinction between mixed-breed and non-mixed-breed dogs. These variables were available for descriptive screening and interpretation but were not used to construct an individual-dog prediction model because the primary analytical unit was the municipality-year.

Municipality-year variables included annual dog admissions, maximum shelter capacity, staff size, total annual budget, rescue-related budget, and veterinary medical budget. Annual resident population, land area, population density, and municipal fiscal self-reliance for 2016–2025 were compiled to describe potential contextual confounding. Population density was treated as a descriptive proxy for urbanization and owner-reclamation or adoption demand, and fiscal self-reliance as a descriptive proxy for local fiscal capacity. These indicators were summarized descriptively and examined in an exploratory sensitivity model, but were not included comprehensively in the primary models because only nine municipalities were available and the indicators were correlated with operator category and municipality identity.

### 2.6. Statistical Analysis

Descriptive analyses summarized dog-level characteristics, shelter outcomes, municipality-year operational variables, and municipality-level background indicators across operator types. Categorical variables were summarized as counts and percentages. Continuous variables were summarized using measures appropriate to their distributions, including medians and interquartile ranges for right-skewed LOS. To evaluate whether overall LOS differences reflected different disposition pathways, LOS was additionally summarized after stratification by final outcome and operator type, with descriptive emphasis on dogs that were adopted or euthanized. LOS values used in tabular summaries were untruncated; values above 365 days were truncated for display only in figures. Final-disposition outcome counts and LOS-specific counts were therefore not forced to be identical, because dogs with confirmed outcomes but missing or invalid date intervals were retained in final-outcome analyses but excluded from LOS-specific summaries. Because LOS was right-skewed and operator type was nested within municipality, these subgroup analyses were interpreted as descriptive and hypothesis-generating.

The main inferential analysis used binomial generalized linear models with a logit link. The municipality-year was selected as the analytical unit because operator category and operational covariates were defined at the municipality or municipality-year level, and all dogs within a municipality-year shared that exposure context. Treating the 33,875 dog admissions as independent units for operator-category inference would have overstated the independent information available. For each municipality-year, the numerator was the number of dogs with the outcome of interest, and the denominator was the number with confirmed final dispositions. Separate models were fit for live release, adoption, RTO, natural mortality, and euthanasia.

For municipality *j* in year *t*, the model was specified as follows:

$$Y_{jt}∼Binomial(n_{jt},p_{jt})$$

$$logit(p_{jt})=log[p_{jt}/(1-p_{jt})]\;logit(p_{jt})=\beta_{0}+\beta_{1}V_{j}+\beta_{2}I_{j}+\beta_{3}{Year}_{t}+\beta_{4}log({Admissions}_{jt})$$

In this model, $Y_{jt}$ denotes the number of dogs with the outcome of interest in municipality j during year t, $n_{jt}$ denotes the number with confirmed final dispositions in that municipality-year, and $p_{jt}$ denotes the outcome probability. $V_{j}$ and $I_{j}$ are indicators for veterinary hospital-operated and individually operated shelters, respectively. ${Year}_{t}$ is calendar year centered at 2016, and ${Admissions}_{jt}$ is annual admissions; the latter enters the model as its natural logarithm.

Municipality fixed effects were not included because operator category was perfectly collinear with municipality identity; fixed effects would absorb all variation used to estimate operator contrasts. A mixed-effects model could be fitted formally, but with only nine municipalities and three municipalities per category, between-municipality variance and operator contrasts would remain weakly identified and sensitive to distributional assumptions. The selected municipality-year model therefore provided transparent point estimates, while CR2 small-cluster inference and deletion analyses were used to characterize uncertainty and composition sensitivity. The coefficients represent exploratory ecological differences among municipal systems, not within-municipality changes or causal operator effects.

Analyses were conducted and documented for reproducibility using Python 3.13.5 with NumPy 2.3.5, SciPy 1.17.0, statsmodels 0.14.6, and Matplotlib 3.10.8. Point estimates were obtained from the municipality-year binomial logit models. Because annual observations were repeated within municipalities, primary uncertainty was estimated using CR2 bias-reduced cluster-robust covariance clustered by municipality, with coefficient-specific Satterthwaite degrees of freedom [30,31]. This approach treats the nine municipalities, rather than the 90 annual rows, as independent clusters. Pearson-scaled quasibinomial covariance was retained as an overdispersion diagnostic and sensitivity analysis but not as the principal inferential basis because it does not explicitly account for within-municipality dependence. The results are reported as odds ratios (ORs), CR2 95% confidence intervals (CIs), Satterthwaite degrees of freedom, and two-sided *p*-values. Robustness analyses included leave-one-municipality-out refitting, a dedicated leave-Andong-out analysis, alternative classifications of unresolved records, beta-binomial logit models, and an exploratory contextual model adjusted for annual fiscal self-reliance and annual log population density. For the fixed 180-day analysis, the same municipality-year logit specification was applied to all admissions eligible for 180 days of potential follow-up, using log-eligible annual admissions as the volume covariate. CR2 covariance and Satterthwaite degrees of freedom were calculated identically to the complete-case models. Sensitivity analyses substituted full annual admissions and excluded the single known-outcome record with an internally inconsistent negative date interval. With nine clusters, all inferential analyses were considered exploratory. Generative artificial intelligence supported language editing, manuscript restructuring, response drafting, checklist review, and code-implementation review; all suggestions were manually verified against source materials and deterministic outputs.

## 3. Results

### 3.1. Study Population and Data Filtering

The source database comprised 40,000 individual animal-management records from the nine municipalities. Of these, 33,878 records were identified as dogs, and 33,875 were eligible admissions during 2016–2025. At the data-closure date, 1037 records lacked a confirmed final disposition because the dog was still in care or the outcome was missing, inconsistent, or unclassifiable. The primary final-disposition cohort therefore consisted of 32,838 dogs (96.9% of eligible admissions; Table 1). This cohort describes confirmed final dispositions among resolved records and should not be interpreted as complete lifetime follow-up for every admission.

### 3.2. Municipality Operational Characteristics

The nine study municipalities were evenly distributed across the three operator categories, with three municipalities in each. The panel contained 90 municipality-year rows but only nine independent municipality clusters. Table 2 provides a compact operator-category summary; detailed municipality-level contextual indicators are reported in Supplementary Table S5. Veterinary hospital-operated shelters accounted for 19,456 admissions, corporation-operated shelters for 7697, and individually operated shelters for 6722. Pohang-si alone contributed 10,021 veterinary hospital-operated admissions (51.5%), so veterinary hospital-operated category summaries were disproportionately influenced by one municipality.

Municipal composition was also heterogeneous within operator categories. Andong-si had the largest mean capacity (404.0 dogs), the highest mean budget per intake (1375.6 thousand KRW), and 522 unresolved admissions, equal to 31.1% of its admissions and 71.7% of all unresolved records from corporation-operated shelters. Among its resolved records, live release comprised 95.7%, and euthanasia comprised 0.9%. Goryeong-gun had the smallest mean capacity (16.4 dogs). These contrasts show that operator category coincided with differences in municipal scale, urbanization, fiscal capacity, infrastructure, and record completion; the detailed values are retained in Supplementary Table S5.

### 3.3. Dog Outcomes by Operator Type

**Figure 1:**
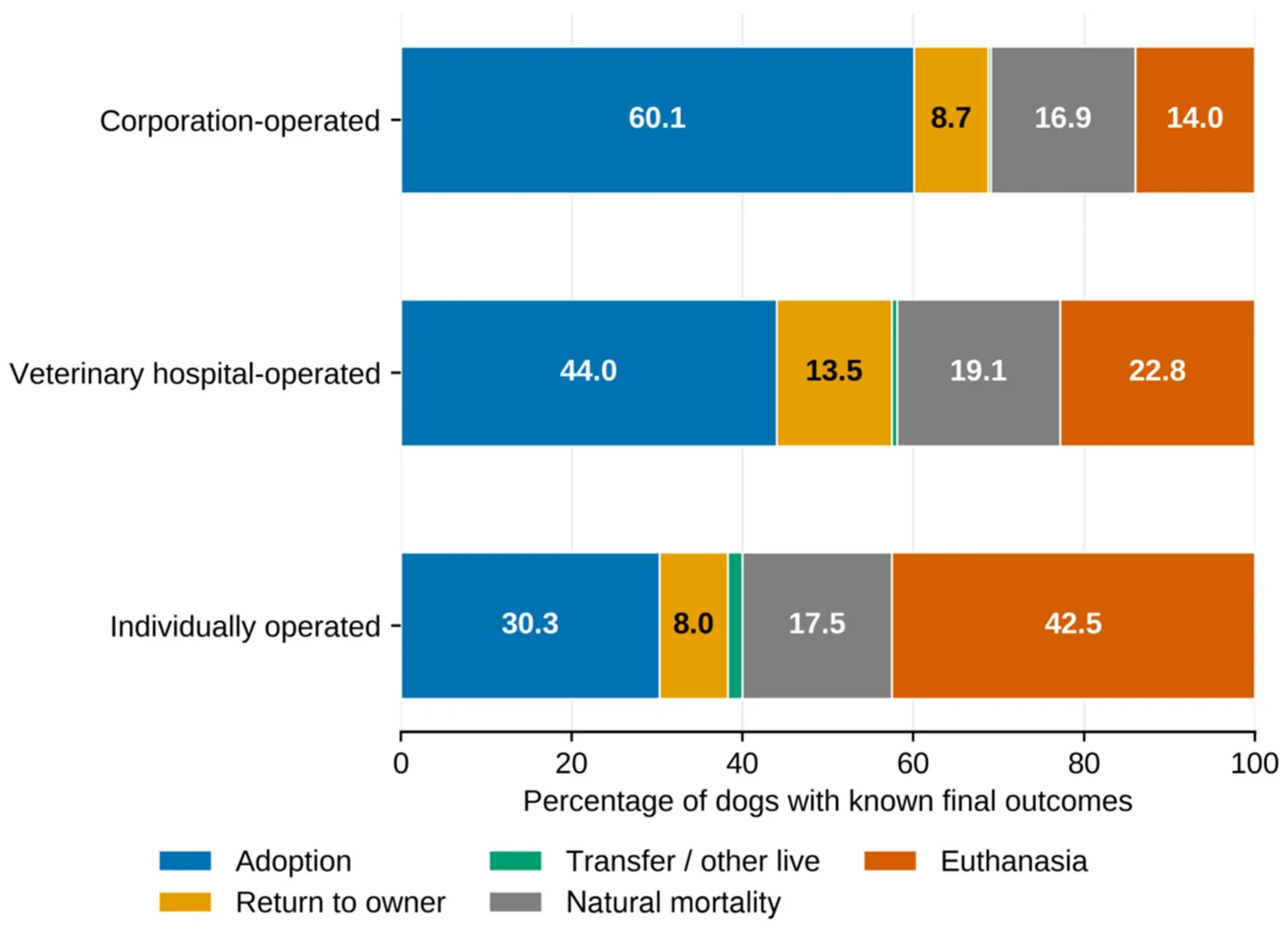
Distribution of final outcomes by contracted operator category. Percentages were calculated among admissions with a known final outcome. The corporation-operated category includes non-profit corporations or animal welfare organizations; the veterinary hospital-operated category includes animal hospitals or veterinary clinics; and the individually operated category includes natural persons.

In this final-disposition analysis, outcome distributions differed descriptively across operator categories. Live release rates were 69.1%, 58.1%, and 40.0% in corporation-operated, veterinary hospital-operated, and individually operated shelters, respectively; euthanasia rates were 14.0%, 22.8%, and 42.5%. Unresolved admissions were unevenly distributed: 728 (9.5% of admissions) in corporation-operated shelters, 299 (1.5%) in veterinary hospital-operated shelters, and 10 (0.1%) in individually operated shelters. Under alternative classifications of unresolved records from corporation-operated shelters, the live-release estimate ranged from 62.6% to 72.0%. Excluding Andong-si reduced the corporation’s complete-case live-release rate to 63.8%, increased euthanasia to 16.6%, and reduced the unresolved proportion to 3.4% (Table 3). Figure 1 displays the corresponding distribution of known final outcomes across operator categories.

**Figure 2:**
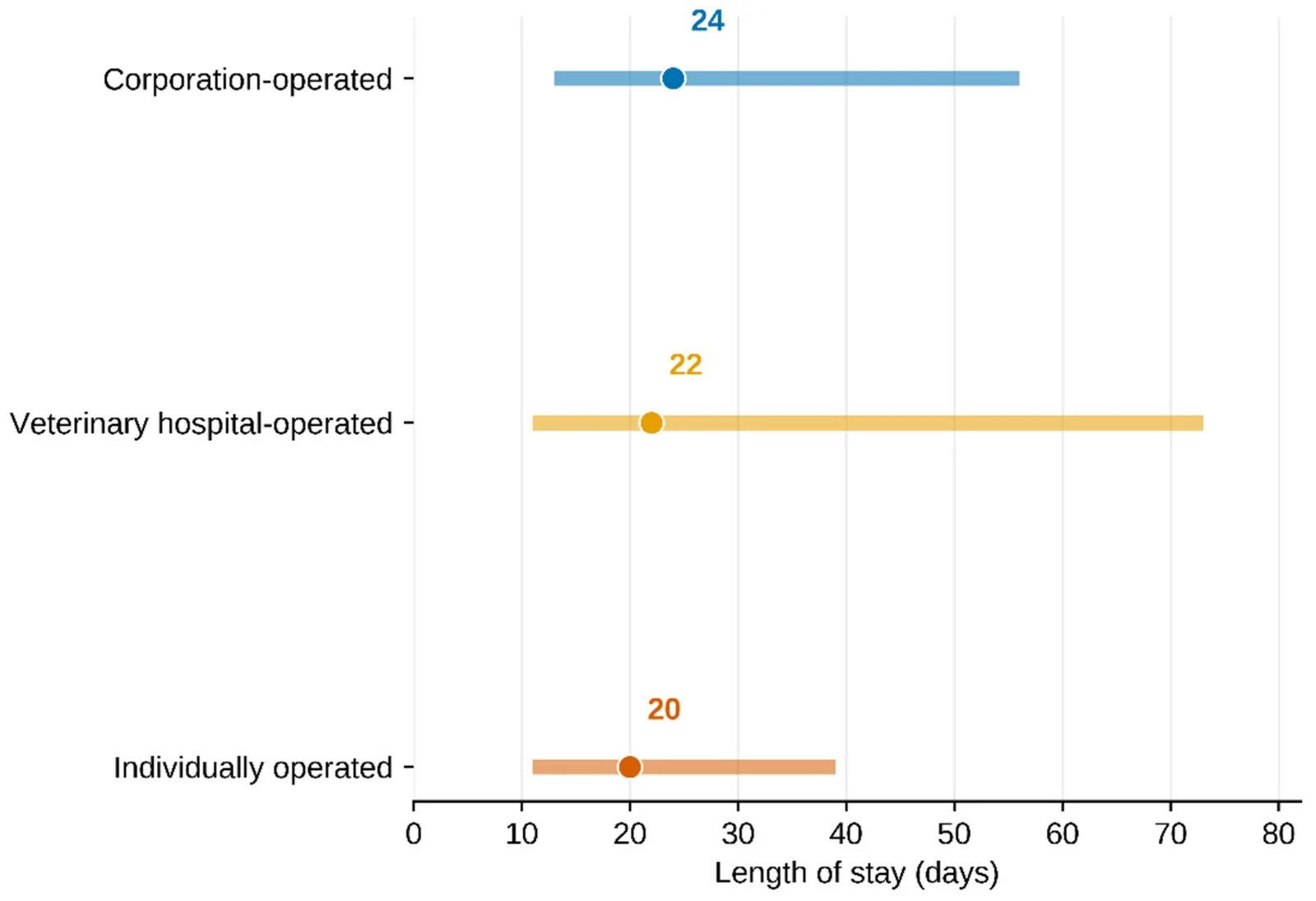
Overall length of stay by contracted operator type. Points show medians and horizontal lines show interquartile ranges, calculated from untruncated records with valid admission and final-disposition dates.

Median LOS differed descriptively across operator categories: 24 days in corporation-operated shelters, 22 days in veterinary hospital-operated shelters, and 20 days in individually operated shelters (Table 3; Figure 2). The shorter median in individually operated shelters should not be interpreted as superior turnover because it coincided with a higher euthanasia proportion. Among euthanized dogs, median LOS was 57, 82, and 32 days, respectively, and 46.7% of euthanized dogs in individually operated shelters had LOS of 30 days or less, compared with 25.4% and 24.7% in the other two categories (Table 4; Supplementary Figure S2). These are empirical disposition and LOS patterns; explanations involving capacity or resource pressure were not tested.

**Table 4:** Length of stay, stratified by selected final disposition and contracted operator type.

Operator Type	Adoption LOS *n*	Adoption LOS Median (IQR), d	Euthanasia Valid LOS *n*	Euthanasia LOS Median (IQR), d	Euthanasia LOS ≤ 30 d, %
Corporation-operated	4191	21 (13–43)	977	57 (30–284)	25.4
Veterinary hospital-operated	8420	22 (12–62)	4372	82 (31–187)	24.7
Individually operated	2033	13 (9–25)	2853	32 (18–59)	46.7

Note: LOS summaries use valid admission and final-disposition dates and untruncated LOS values. The euthanasia valid LOS *n* is LOS-specific; one veterinary hospital-operated euthanasia record lacked a valid LOS interval, so the LOS-specific total is 8202 whereas the final-disposition euthanasia total in Table 5 and Table S4 is 8203. The ≤ 30 d column is calculated among euthanized dogs with valid LOS. IQR = interquartile range; LOS = length of stay.

**Table 5:** Municipality-year binomial logit models with CR2 municipality-clustered inference.

Outcome	*n*/*N*	Veterinary Hospital-Operated OR (95% CI); df	*p*	Individually Operated OR (95% CI); df	*p*	Year OR (95% CI); df	*p*
Live release	18,638/32,838	0.52 (0.05–5.44); df 3.25	0.456	0.29 (0.02–3.76); df 3.66	0.242	1.08 (0.94–1.24); df 4.72	0.195
Adoption	14,644/32,838	0.43 (0.06–2.99); df 3.19	0.269	0.28 (0.03–2.52); df 3.67	0.178	1.07 (0.93–1.22); df 4.68	0.270
Return to owner	3724/32,838	1.43 (0.34–5.94); df 3.06	0.490	0.93 (0.12–6.88); df 3.60	0.916	1.02 (0.96–1.08); df 3.91	0.418
Natural mortality	5997/32,838	1.59 (0.37–6.80); df 3.31	0.399	1.03 (0.25–4.26); df 3.75	0.958	0.94 (0.80–1.11); df 4.82	0.377
Euthanasia	8203/32,838	1.66 (0.17–16.32); df 3.38	0.548	4.72 (0.40–56.35); df 3.21	0.145	0.95 (0.83–1.08); df 4.69	0.321

Note: Models used 90 municipality-year rows nested within nine municipality clusters and adjusted for calendar year centered at 2016 and log annual dog intake. Confidence intervals and *p*-values use CR2 bias-reduced cluster-robust covariance with coefficient-specific Satterthwaite degrees of freedom. Corporation-operated shelters were the reference category. Because the operator category was perfectly nested within the municipality identity, estimates represent exploratory between-system associations. CI = confidence interval; OR = odds ratio; RTO = return to owner.

Annual complete-case live-release and euthanasia rates are provided in Supplementary Figures S3 and S4 and Table S1 rather than in the main text. These descriptive curves were not interpreted as cohort trends because follow-up completeness differed by intake year and operator category. The concern was most evident for corporation-operated shelters in 2025, when 227 of 759 admissions (29.9%) remained unresolved while live release among the 532 resolved records was 78.8%. The reviewer-requested fixed 180-day analysis was therefore used to assess whether the direction of the estimated year effect persisted after equalizing potential follow-up.

### 3.4. Municipality-Year Regression Analyses

Municipality-year logit point estimates were unchanged, but CR2 municipality-clustered inference materially increased uncertainty. Compared with corporation-operated shelters, the live-release OR was 0.52 for veterinary hospital-operated shelters (CR2 95% CI, 0.05–5.44; df = 3.25; *p* = 0.456) and 0.29 for individually operated shelters (95% CI, 0.02–3.76; df = 3.66; *p* = 0.242). Adoption contrasts were 0.43 (95% CI, 0.06–2.99) and 0.28 (95% CI, 0.03–2.52), respectively (Table 5). These are exploratory municipality-year associations, not estimated causal effects of operator category.

For RTO, the veterinary hospital-operated versus corporation-operated point estimate was 1.43 (CR2 95% CI, 0.34–5.94), and the individually operated versus corporation-operated estimate was 0.93 (95% CI, 0.12–6.88). For euthanasia, the corresponding ORs were 1.66 (95% CI, 0.17–16.32; df = 3.38; *p* = 0.548) and 4.72 (95% CI, 0.40–56.35; df = 3.21; *p* = 0.145). All operator-category CR2 intervals crossed the null. The point estimates describe the direction and magnitude observed among these nine municipal systems, but they do not provide statistically precise confirmation of an independent operator effect.

### 3.5. Fixed 180-Day Outcome Analysis

The common data closure date was 25 June 2026. Admissions on or before 27 December 2025 had 180 days of potential follow-up; 33,836 admissions met this criterion, and 39 admissions from 28 to 31 December 2025 were excluded. By day 180, 17,576 dogs (51.9%) were released alive, and 6765 (20.0%) were euthanized. By operator category, live release comprised 59.9% in corporation-operated systems, 53.0% in veterinary hospital-operated systems, and 39.8% in individually operated systems; euthanasia comprised 9.1%, 16.7%, and 42.0%, respectively (Table 6; Supplementary Tables S9–S11 and Figure S5).

In fixed-window CR2 models, the live-release OR was 0.53 for veterinary hospital-operated versus corporation-operated systems (95% CI, 0.06–5.03; df = 3.30; *p* = 0.449) and 0.45 for individually operated versus corporation-operated systems (95% CI, 0.05–4.40; df = 3.77; *p* = 0.377). The corresponding euthanasia ORs were 1.75 (95% CI, 0.34–8.95; df = 3.65; *p* = 0.384) and 7.76 (95% CI, 0.83–72.27; df = 3.17; *p* = 0.062). All fixed-window operator-contrast intervals crossed the null, consistent with the small number of independent municipalities (Supplementary Table S12).

The live-release year OR changed from 1.08 in the complete-case model (95% CI, 0.94–1.24; *p* = 0.195) to 1.05 in the fixed 180-day model (95% CI, 0.93–1.19; df = 5.00; *p* = 0.344). Thus, the positive point direction persisted but attenuated and remained statistically imprecise. The euthanasia year OR changed from 0.95 (95% CI, 0.83–1.08; *p* = 0.321) to 0.90 (95% CI, 0.78–1.05; df = 4.82; *p* = 0.130). Using full annual admissions as the volume covariate or excluding the internally inconsistent date record did not materially alter these estimates.

### 3.6. Robustness and Sensitivity Analyses

Pearson dispersion factors were greater than 1 for all municipality-year outcome models: 48.32 for live release, 36.43 for adoption, 12.21 for RTO, 32.12 for natural mortality, and 38.15 for euthanasia. These diagnostics confirmed substantial extra-binomial dispersion, but Pearson scaling alone did not account for dependence among annual observations from the same municipality. CR2 municipality-clustered inference therefore served as the principal small-cluster uncertainty analysis, while Pearson-scaled and beta-binomial results were retained as sensitivity estimates in Supplementary Table S2.

Leave-one-municipality-out refitting retained the direction of the main live-release, adoption, and euthanasia point estimates, but the magnitudes were sensitive to sample composition. For live release, veterinary hospital-operated versus corporation-operated ORs ranged from 0.20 to 0.72 and individually operated versus corporation-operated ORs from 0.12 to 0.39. For euthanasia, veterinary hospital-operated versus corporation-operated ORs ranged from 1.15 to 4.88 and individually operated versus corporation-operated ORs from 3.39 to 12.70. The direction of the individually operated versus corporation-operated euthanasia association was stable, but its magnitude was highly sensitive to which municipality was omitted and should not be described as a stable effect size.

The contextual sensitivity analysis was particularly important for interpretation. After adjustment for annual fiscal self-reliance and log population density, the veterinary hospital-operated versus corporation-operated OR attenuated to 0.97 for both live release and euthanasia, whereas the individually operated versus corporation-operated ORs were 0.23 and 5.92. Because only nine municipalities were available and the contextual covariates were correlated with operator category, these values are diagnostic rather than confirmatory; they indicate that the veterinary hospital-operated contrast was highly context-sensitive. A dedicated leave-Andong-out analysis changed the hospital- and individually operated versus corporation-operated live-release ORs from 0.52 and 0.29 to 0.59 and 0.39, and the euthanasia ORs from 1.66 and 4.72 to 1.48 and 3.81. CR2 intervals became especially wide because only two corporation-operated municipalities remained (Supplementary Tables S7 and S8).

Unresolved records were substantively important: 522 of 728 unresolved corporation-operated admissions originated from Andong-si. Figure S1 presents live release, euthanasia, and unresolved proportions for each municipality using denominator-specific panels. The administrative records did not identify why a case remained unresolved, so this concentration cannot be used as evidence of a low-euthanasia or no-kill management philosophy. Still-in-care records were right-censored at data closure, and complete-case rates depended on follow-up duration. In the fixed-window analysis, all eligible admissions remained in the denominator, and records without a documented live release, euthanasia, or natural death by day 180 were classified as having no documented final disposition by day 180. This reduced differential follow-up bias but did not remove ecological confounding or composition sensitivity.

## 4. Discussion

### 4.1. Principal Findings

This 10-year retrospective ecological study found descriptive differences in recorded outcomes across operator-linked municipal systems in three operator categories. Among dogs with confirmed final dispositions, corporation-operated shelters had the highest live release and adoption rates, veterinary hospital-operated shelters the highest RTO rates, and individually operated shelters the highest euthanasia rates. The fixed-window results retained the same broad ordering: live release by day 180 was 59.9%, 53.0%, and 39.8%, whereas euthanasia by day 180 was 9.1%, 16.7%, and 42.0%, respectively. These are the direct empirical findings. They occurred alongside substantial differences in unresolved follow-up, intake volume, population density, fiscal context, capacity, staffing, and municipality composition.

The inferential evidence remained imprecise in both the complete-case and fixed-window analyses. All CR2 intervals for complete-case operator contrasts included the null, reflecting only nine independent municipalities and three municipalities per category; all fixed-window operator intervals also crossed the null. The complete-case live-release year OR of 1.08 attenuated to 1.05 in the fixed 180-day model (95% CI, 0.93–1.19; *p* = 0.344), so equal potential follow-up did not yield statistically precise evidence of a temporal trend. Leave-one-municipality-out analyses retained several directions but showed marked magnitude variation, including an individually operated versus corporation-operated euthanasia OR range of 3.39–12.70. The regression estimates are therefore hypothesis-generating municipality-year associations rather than confirmatory operator effects.

Operator category was perfectly nested within municipality identity and functioned as a marker of a wider service configuration. Population density, fiscal capacity, contract provisions, facility quality, disease burden, animal-registration practices, adoption and rescue networks, enforcement, and service demand may all contribute to the observed patterns. Contextual adjustment attenuated the veterinary hospital-operated versus corporation-operated ORs to 0.97 for both live release and euthanasia, and Pohang-si and Andong-si materially influenced their respective categories. The study cannot determine whether an individual dog would have had a different outcome under another operator.

### 4.2. Interpretation of Operator-Type Patterns

Empirically, corporation-operated shelters had the highest complete-case live-release and adoption rates. Structured administration, adoption promotion, and volunteer or rescue networks are possible explanations, but these features were not measured and remain hypotheses rather than demonstrated mediators. Interpretation is also affected by 728 unresolved records from corporation-operated shelters, including 522 from Andong-si. The corporation live-release estimate ranged from 62.6% to 72.0% under alternative assumptions and decreased to 63.8% when Andong-si was excluded; the 69.1% complete-case estimate therefore does not establish a uniformly superior operator category.

Empirically, veterinary hospital-operated shelters had the highest RTO rate. Microchip scanning, registration verification, owner contact, and clinical triage are plausible mechanisms in the Korean statutory pathway [2,32,33], but these processes were not measured. Pohang-si accounted for more than half of admissions in this category, and the veterinary hospital-operated versus corporation-operated live-release and euthanasia ORs both attenuated to 0.97 after exploratory contextual adjustment. The hospital profile should therefore be understood as a context-dependent observation, not evidence that hospital operation independently improves owner reclamation.

Empirically, individually operated shelters had the lowest complete-case live-release rate, the highest euthanasia rate, a 32-day median euthanasia LOS, and 46.7% of euthanized dogs being disposed of within 30 days. Capacity, staffing, veterinary access, fiscal constraints, intake case mix, rurality, and contract design are plausible explanations, but none was established as a mechanism in this study. The pattern identifies a prospective audit priority; it does not show that the individual operation itself caused rapid euthanasia or that changing operator category would remove the pattern.

Variation within each category further shows that the operator label alone is an incomplete explanation. Future studies should distinguish the empirical outcomes reported here from hypothesized mediators by measuring contract terms, facility conditions, staffing, budget execution, veterinary access, adoption demand, transfer networks, and decision processes directly. Multi-level longitudinal designs with more municipalities are needed to separate dog-level case mix, municipal context, and operator arrangements.

### 4.3. Methodological Considerations

The primary denominator produced a final-disposition analysis at data closure. Restricting rates to confirmed outcomes ensures internally consistent disposition categories but can bias absolute rates when unresolved records are unevenly distributed. This concern was greatest in the corporation-operated group and later cohorts; in 2025, 29.9% of corporation-operated admissions were unresolved. The fixed 180-day analysis addressed this problem by retaining all 33,836 admissions with equal potential follow-up; 39 late admissions were excluded. The live-release year point direction persisted but attenuated from 1.08 to 1.05, and its CR2 interval crossed 1. The euthanasia year OR was 0.90 and also crossed 1. Unequal potential follow-up was therefore not sufficient to reverse the live-release coefficient’s point direction, but the fixed window did not establish a temporal trend. The binary endpoint does not capture outcomes occurring after day 180 and is not a complete lifetime outcome; competing-risk survival analysis would provide additional detail.

The effective inferential sample was small. Ninety municipality-year rows represented repeated observations within only nine municipalities, and operator category did not vary within municipality. CR2 covariance with Satterthwaite degrees of freedom addressed clustered uncertainty more appropriately than Pearson scaling alone, but no variance estimator or mixed-effects specification can replace the independent information absent when only three municipalities represent each category. The analysis remains exploratory and underpowered for precise municipality-level contrasts.

The ecological design also leaves residual confounding and precludes dog-level inference. Population density and fiscal self-reliance were examined, but unmeasured differences in contracts, facilities, local animal populations, service demand, case severity, and community adoption infrastructure may explain part of the observed gradient. Breed, body size or weight, and intake health status were not incorporated into the municipality-year models, and standardized behavior, microchip status, reproductive status, disease severity, prior ownership, and detailed euthanasia reasons were not consistently available. A municipality-level association cannot be assumed to apply to an individual dog.

### 4.4. Veterinary Public Health and Policy Implications

The empirical results support priorities for measurement and monitoring, not the effectiveness of a particular intervention. The recommendations below are derived from current Korean law, shelter-medicine guidance, and public-administration frameworks rather than from causal effects estimated in this study. Candidate monitoring domains include outcome completeness, explicit data-closure dates, fixed follow-up periods, intake pressure, capacity, staffing, veterinary access, owner reclamation, adoption pathways, and euthanasia review.

Current Korean law and the Animal Protection Center Operating Guidelines assign municipalities responsibility for rescue, protection, owner notification, shelter designation, monitoring, and humane disposition [2,34]. Consistent with the Association of Shelter Veterinarians’ guidance [35], municipalities could pilot standardized record-keeping, minimum-care protocols, and transparent euthanasia review. Longitudinal intake-and-outcome reporting [36] and capacity-for-care approaches [37,38] further support triggers that link intake to housing, staff time, veterinary availability, foster or transfer capacity, and adoption activity. These are prospective policy options; their effectiveness must be evaluated with pre-specified outcomes and equal follow-up rather than inferred from operator-category differences.

Owner reclamation and adoption systems also require direct measurement. Lost-animal identification, public search tools, adoption and relinquishment interventions, and post-adoption support may influence outcomes [32,33,39,40], but their role in Korean contracted shelters remains uncertain. Public-administration evidence likewise suggests that service quality depends on contract design, monitoring, value alignment, government-contractor relationships, and stakeholder co-production [21,22,23,24,25,26]. Accordingly, monitoring, capacity planning, and standardized municipal support should be evaluated across operator arrangements rather than assuming that replacement of one operator category with another will improve welfare.

### 4.5. Limitations

First, this was a regional ecological study of nine municipalities in one province, with only three municipalities per operator category. The 90 annual rows did not constitute 90 independent units, CR2 intervals were broad, alternative multi-level specifications remained weakly identified, and the findings are not nationally representative.

Second, the operator category was non-random, time-invariant, and perfectly nested within municipality identity. The models could not separate operator category from contract terms, facility quality, disease burden, animal-registration practices, public awareness, adoption demand, enforcement, rurality, or other stable municipal characteristics. Pohang-si disproportionately influenced the veterinary hospital-operated category, and Andong-si was atypical within the corporation-operated category, making category summaries composition-sensitive.

Third, residual dog-level case-mix and measurement limitations remain. Several individual descriptors were unavailable or not modeled, outcome coding may have differed among municipalities, and unresolved records were highly uneven. The fixed 180-day endpoint reduced unequal potential follow-up but depended on administrative date accuracy, did not capture events after day 180, and contained one known-outcome record with an internally inconsistent negative interval. Primary coding treated that record as no documented event by day 180, and an exclusion sensitivity analysis was materially unchanged. Reasons for unresolved status and later events were not fully observed.

Fourth, leave-one-municipality-out analyses showed that directional stability did not imply a stable effect magnitude. The study included dogs only and should not be extrapolated to other species. Collectively, these limitations restrict the permissible inference to descriptive, operator-linked municipal patterns; they preclude causal conclusions, individual-dog predictions, and claims that one contracting model is generally superior.

## 5. Conclusions

Across nine municipalities in Gyeongsangbuk-do, final-disposition distributions differed descriptively among corporation-operated, veterinary hospital-operated, and individually operated contracted shelters. Corporation-operated shelters had the highest complete-case live-release rate, veterinary hospital-operated shelters the highest RTO rate, and individually operated shelters the highest euthanasia rate. A fixed 180-day analysis retained the broad live-release and euthanasia ordering while applying equal potential follow-up. These patterns occurred within different municipal, organizational, fiscal, and data-completeness contexts. They do not establish causal operator effects, rank contracting models, or show that changing operator category alone would improve welfare. Future comparisons should use standardized outcome definitions, equal follow-up windows, complete reporting, and prospective designs across more municipalities.

Only nine municipalities contributed independent information; all complete-case and fixed-window CR2 operator-contrast intervals crossed the null, leave-one-municipality-out estimates varied substantially in magnitude, and contextual adjustment attenuated the veterinary hospital-operated contrasts. The fixed-window live-release year OR was 1.05 (95% CI, 0.93–1.19; *p* = 0.344), indicating that the positive point direction persisted but did not establish a temporal trend. The most defensible conclusion is that operator labels marked broader municipal service configurations in this regional sample. Evidence-aligned priorities are stronger outcome monitoring, explicit data-closure dates, fixed post-intake follow-up, capacity planning, standardized municipal support, direct measurement of contract and facility conditions, and prospective evaluation across a broader multi-region sample.

## Figures and Tables

**Table 1:** Study population and data selection flow.

Data-Selection Step	Number of Records
All source animal-record entries	40,000
Dog records identified from municipal ledgers	33,878
Dog admissions within 2016–2025	33,875
Dog admissions with known final outcomes	32,838
Records still in care, missing, or unclassified	1037
Dog records outside 2016–2025 excluded from primary analysis	3

Note: Dogs were identified from the species field in the municipal ledgers. Known-final-outcome denominators exclude records still in care, missing, ambiguous, or unclassified at data closure; the resulting analyses are final-disposition analyses rather than complete lifetime-outcome analyses.

**Table 2:** Summary of operator categories by municipal demographic, fiscal, and operational context, 2016–2025.

Operator Category	Admissions	Mean Population	Density/km^2^	Mean Capacity, Dogs	Mean Staff, Persons	Budget/Intake, 1000 KRW	Fiscal Self-Reliance, %
Corporation-operated	7697	107,025	145.4	191.7	2.6	659.9	16.98
Veterinary hospital-operated	19,456	287,645	367.3	162.0	3.9	455.1	20.95
Individually operated	6722	51,062	67.6	28.4	1.8	267.8	13.42

Note: Each operator category contained three municipalities. Admissions are category totals; the remaining values are unweighted means of municipality-level summaries for 2016–2025. Mean budget per intake is based on the annual total budget divided by the annual dog intake. Detailed municipality-specific values, fiscal ranges, and data definitions are provided in Supplementary Table S5.

**Table 3:** Outcome distribution by type of contracted operator.

Operator Type	Admissions	Known Final	Unresolved *n* (%)	Live Release %	Adoption %	Return %	Natural Mortality %	Euthanasia %	LOS Median (IQR), d
Corporation-operated	7697	6969	728 (9.5)	69.1	60.1	8.7	16.9	14.0	24 (13–56)
Veterinary hospital-operated	19,456	19,157	299 (1.5)	58.1	44.0	13.5	19.1	22.8	22 (11–73)
Individually operated	6722	6712	10 (0.1)	40.0	30.3	8.0	17.5	42.5	20 (11–39)
Corporation-operated excluding Andong (sensitivity)	6016	5810	206 (3.4)	63.8	58.0	5.5	19.6	16.6	-

Note: Live release, adoption, return, natural mortality, and euthanasia use known final outcomes as the denominator; unresolved percentages use all admissions. The corporation-operated row excluding Andong is a sensitivity analysis, not a fourth operator category; LOS could not be recomputed from the aggregated revision dataset. Live release includes adoption, return to owner, transfer, and other documented live outcomes. IQR = interquartile range; LOS = length of stay.

**Table 6:** Fixed 180-day outcomes by contracted operator category.

Operator Category	Eligible Admissions	Live Release by Day 180, *n* (%)	Euthanasia by Day 180, *n* (%)	Natural Mortality by Day 180, *n* (%)	No Documented Final Disposition by Day 180, *n* (%)
Corporation-operated	7678	4598 (59.9%)	700 (9.1%)	926 (12.1%)	1454 (18.9%)
Veterinary hospital-operated	19,438	10,301 (53.0%)	3241 (16.7%)	3377 (17.4%)	2519 (13.0%)
Individually operated	6720	2677 (39.8%)	2824 (42.0%)	1162 (17.3%)	57 (0.8%)

Note: Eligible admissions had at least 180 days of potential follow-up at the common data-closure date (intake on or before 27 December 2025). The denominator is all eligible admissions. “No documented final disposition by day 180” includes records unresolved at day 180 and terminal events documented after day 180. One veterinary hospital-operated record had an internally inconsistent disposition date; treating it as no event or excluding it produced materially unchanged estimates.

## Data Availability

The supplementary workbook and figure files provide aggregated municipality-year datasets, the de-identified fixed 180-day municipality-year analytic panel, annual fiscal and population-density context tables, model-output tables, robustness analyses, stratified LOS summaries, and supplementary figures where permitted by the data-providing institutions; analysis code is not included. Row-level raw municipal administrative data are not publicly deposited in a repository, but may be made available by the corresponding author upon reasonable request, subject to authorization by the relevant municipalities and applicable legal, administrative, and privacy-related data-use restrictions. Requests to access the row-level raw data should be directed to Byeong-Cheol Song, sbc07016@korea.kr. Where full row-level records cannot be released, a de-identified minimal analytic dataset may be provided under approved data-use conditions.

## Supplementary Materials

The following supporting information can be downloaded at: https://www.mdpi.com/article/10.3390/ani16172657/s1. Supplementary Tables S1–S12 are contained in the supplementary workbook, and Supplementary Figures S1–S5 are supplied as separate high-resolution image files with a separate supplementary figure-legend document: Table S1: Annual complete-case operator-level outcome rates; Table S2: Pearson-scaled quasibinomial, beta-binomial, and CR2 operator-contrast estimates; Table S3: Leave-one-municipality-out summary; Table S4: Sensitivity analyses for unresolved records; Table S5: Detailed municipality demographic, fiscal, and operational indicators; Table S6: LOS by final disposition and operator category; Table S7: Exploratory contextual sensitivity models; Table S8: Dedicated leave-Andong-out model sensitivity; Table S9: Municipality-level fixed 180-day outcomes; Table S10: Operator-level fixed 180-day outcomes; Table S11: Annual fixed 180-day outcome rates; Table S12: Fixed 180-day CR2 model results; Figure S1: Municipality-level live release, euthanasia, and unresolved proportions; Figure S2: Stratified LOS summary; Figure S3: Annual complete-case live-release rates; Figure S4: Annual complete-case euthanasia rates; Figure S5: Annual fixed 180-day live-release and euthanasia rates.

## Abbreviations

The following abbreviations are used in this manuscript:

CI	confidence interval
CR2	bias-reduced cluster-robust variance estimator
df	degrees of freedom
IQR	interquartile range
KRW	Korean won
LOS	length of stay
OR	odds ratio
RECORD	REporting of studies Conducted using Observational Routinely-collected health Data
RTO	return to owner
STROBE	Strengthening the Reporting of Observational Studies in Epidemiology
STROBE-Vet	Strengthening the Reporting of Observational Studies in Epidemiology–Veterinary Extension
