# Supplementary material for: Municipal Outsourcing and Animal Welfare Outcomes in Contracted Dog Shelters: A 10-Year Retrospective Policy Evaluation in Korea
Source: Animals (Basel). 2026 Aug 24;16(17):2657. doi: 10.3390/ani16172657 (PMC13564333; doi:10.3390/ani16172657)
Supplement: Supplementary file 1 [file animals-16-02657-s001.zip › Supplementary_Figure_Legends_SUBMISSION_READY.pdf]

Supplementary Figure Legends

Municipal Outsourcing and Animal Welfare Outcomes in Contracted Dog Shelters: A 10-Year Retrospective Policy Evaluation in Korea

Figure S1. Municipality-level live-release, euthanasia, and unresolved proportions by contracted operator type. Live-release and euthanasia panels use dogs with known final outcomes; the unresolved panel uses all admissions. Full municipality labels identify the corresponding operator category, and horizontal separators distinguish the three groups.

Figure S2. Length of stay for adopted and euthanized dogs by contracted operator type. Points show medians and horizontal lines show interquartile ranges; adoption and euthanasia are displayed on separate scales, using untruncated records with valid admission and final-disposition dates.

Figure S3. Annual complete-case live-release rates by contracted operator category, 2016–2025. Percentages use dogs with known final outcomes; intake cohorts did not share a common post-intake follow-up window.

Figure S4. Annual complete-case euthanasia rates by contracted operator category, 2016–2025. Percentages use dogs with known final outcomes; intake cohorts did not share a common post-intake follow-up window.

Figure S5. Annual fixed 180-day live-release and euthanasia rates by contracted operator category, 2016–2025. Percentages use all admissions with 180 days of potential follow-up by the common data-closure date of 25 June 2026; 39 admissions after 27 December 2025 were excluded.
